# The Role of NLRP1, AIM2 and MEFV Inflammasomes in the High‐Intensity Interval Training of Individuals With Obesity

**DOI:** 10.1111/imm.70090

**Published:** 2025-12-21

**Authors:** Ana Luíza Pereira Assunção Silveira, Daniela Alves de Abreu, Amanda de Lima Santos Musto, Luiz Henrique da Silva Nali, Jônatas Bussador do Amaral, André Luis Lacerda Bachi, Marina Tiemi Shio, Paula Rezende‐Teixeira, Carolina Nunes França

**Affiliations:** ^1^ Post Graduation Program in Health Sciences, Santo Amaro University São Paulo Brazil; ^2^ ENT Research Lab, Department of Otorhinolaryngology—Head and Neck Surgery Federal University of Sao Paulo São Paulo Brazil

**Keywords:** high interval intensity training, inflammasome, obesity

## Abstract

Obesity is a chronic disease associated with systemic inflammation caused by excess visceral fat and pro‐inflammatory cytokines such as IL‐1β and IL‐6. Inflammasomes—particularly those involving genes such as NLRP1, AIM2 and MEFV—play a key role in this process. High‐intensity interval training (HIIT) can counteract this inflammation; however, it remains unclear how HIIT modulates inflammasome gene expression in obesity. This study investigated whether HIIT can alter the expression of genes related to the inflammasomes NLRP1, AIM2 and MEFV in obese individuals. The results showed that, after 8 weeks of HIIT, there was an increase in the expression of the genes AIM2, MEFV, CARD16 and CARD18. The increase in CARD16, known to inhibit caspase‐1 dimerisation, reinforces the hypothesis related to decreased inflammation, evidenced by the absence of clear activation of the NLRP1 inflammasome and by lower serum IL‐1β concentrations in trained participants. Although CARD18 was also upregulated, its function remains ambiguous, and it may act as an inhibitor or modulator of inflammation. Therefore, we conclude that HIIT is a promising intervention for modulating inflammatory genes in individuals with obesity, with the potential to reduce systemic inflammation and its pathological effects.

## Graphical abstract

This study shows that 8 weeks of high‐intensity interval training (HIIT) modulate inflammasome‐related gene expression in individuals with obesity. HIIT increased AIM2, MEFV, CARD16 and CARD18 expression, with CARD16 upregulation supporting reduced inflammation through inhibition of caspase‐1 activation and lower IL‐1β levels. These findings highlight HIIT as a promising strategy to attenuate systemic inflammation linked to obesity.

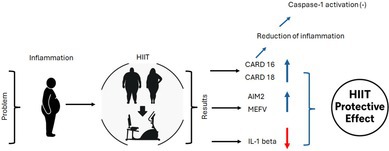

## 1 Introduction

Obesity is a chronic, multifactorial disease influenced by environmental, biological, psychosocial and socioeconomic factors [1]. According to the World Health Organization (WHO), it is characterised by excessive fat accumulation in adipose tissue, which typically contains around 40% of the body's macrophages—a proportion that is closely linked to the extent of tissue inflammation [2].

There is a close relationship between obesity and systemic inflammation, which contributes to disturbances in carbohydrate and lipid metabolism, promoting the development of diseases such as type 2 diabetes and atherosclerosis [3, 4]. This chronic inflammatory state is associated with the activation of intracellular protein complexes known as inflammasomes [5]. Several studies have shown that inflammasomes are involved in the pathogenesis of various diseases, including cancer, diabetes and Alzheimer's disease [6, 7, 8]. Inflammasomes are multiprotein complexes composed of sensor receptors (such as NLRP1, AIM2, MEFV and NLRC3), an adaptor protein called ASC (apoptosis‐associated speck‐like protein containing a CARD), and the enzyme procaspase‐1, that, when activated, triggers the secretion of proinflammatory cytokines, such as interleukin‐1β (IL‐1β) [5].

The NLRP1, AIM2 and MEFV genes play central roles in the activation and regulation of inflammasomes, directly impacting inflammatory responses and tissue homeostasis. NLRP1 responds to intracellular stress signals, such as proteolytic degradation or danger signals [9]. In turn, AIM2 directly recognises exogenous or endogenous cytosolic DNA [10]. Both sensors modulate acute inflammation against pathogens and possibly autoimmune reactions, leading to the release of IL‐1β and IL‐18, and ultimately triggering pyroptotic cell death [9, 10, 11]. MEFV gene encodes the pyrin protein that forms the pyrin inflammasome, this is a sensor of modifications in the activity of RhoA GTPases, a family of proteins involved in cell signalling and cytoskeletal organisation. When bacteria or bacterial toxins inactivate these GTPases, pyrin can become activated and initiate inflammatory responses [12]. On the other hand, the pyrin protein also acts as a negative modulator, controlling excessive inflammasome activation. Mutations in this gene, such as those observed in familial Mediterranean fever, result in overproduction of pro‐inflammatory cytokines and chronic systemic inflammation [10].

Other genes also control the inflammasome activation, such as CARD16 and CARD18. CARD16 and CARD18, members of the Caspase Recruitment Domain (CARD)‐only proteins, known as COPs, are also involved in the negative modulation of inflammation, primarily by acting as inhibitors of caspase‐1 activation [13]. Together, these genes reflect the balance between pro‐ and anti‐inflammatory signals and are targets of growing interest in studies of chronic inflammatory diseases, such as obesity, and in investigations into the regulatory effects of physical exercise [13].

High‐intensity physical exercise has been shown to be an effective strategy in combating chronic inflammation associated with obesity. Individuals with obesity often exhibit a low‐grade inflammatory state, characterised by persistent activation of the innate immune system and the continuous release of pro‐inflammatory cytokines, such as IL‐6, TNF‐α and IL‐1β, mainly originating from visceral adipose tissue [14]. This condition contributes to the development of insulin resistance, endothelial dysfunction and increased cardiometabolic risk [14]. Regular practice of intense exercise, such as high‐intensity interval training (HIIT), can positively modulate this inflammatory state, reducing inflammatory markers, improving insulin sensitivity and promoting beneficial metabolic adaptations [15, 16, 17]. These effects occur, in part, by reducing oxidative stress, improving mitochondrial function and decreasing macrophage infiltration into adipose tissue, demonstrating the potential of high‐intensity exercise as a nonpharmacological intervention in the management of chronic inflammation in individuals with obesity [16].

In the context of obesity, a chronic low‐grade inflammatory state is maintained, in part, by the increased expression of pro‐inflammatory cytokines such as interleukin‐1 alpha (IL‐1α), encoded by the IL‐1A gene [14]. This cytokine acts as a potent inflammatory mediator, promoting the activation of signalling pathways such as NF‐κB and contributing to the perpetuation of the inflammatory environment in adipose tissue [14]. Conversely, genes such as CARD16, CARD18 and MEFV, which encode inflammatory‐inhibitory proteins, play a negative regulatory role by preventing caspase‐1 activation and consequently the maturation of IL‐1β and IL‐18 in inflammasomes [13].

However, a gap still exists in understanding how HIIT modulates the NLRP1 and AIM2 inflammasomes and related genes in individuals with obesity, particularly when compared to untrained counterparts. Previous studies have indicated that multiple genes may regulate inflammasome and that its activation can occur through alternative pathways [18]. Therefore, investigating the effects of HIIT on inflammasome gene expression in this population may not only clarify the regulatory mechanisms underlying inflammasome dynamics and associated genes but also enhance our understanding of the therapeutic potential of HIIT—a nonpharmacological intervention known for its high adherence and health benefits in individuals with obesity.

In this sense, the objectives of this study were to investigate the effects of HIIT on the modulation of the NLRP1, AIM2 and MEFV inflammasomes, as well as regulatory genes associated with the inflammatory response, such as CARD16 and CARD18, in individuals with obesity. The study sought to understand whether HIIT could influence the expression of these genes, promoting changes that might contribute to the regulation of pro‐inflammatory cytokine release, such as IL‐1β. In this way, the aim was to broaden the understanding of the role of intense physical exercise as a nonpharmacological intervention in the control of chronic inflammation associated with obesity, providing insights for the development of more effective and sustainable therapeutic approaches.

## 2 Materials and Methods

### 2.1 Study Design and Sample Size Calculation

This is a prospective, controlled and randomised study involving experimental and control groups to evaluate the effects of 8 weeks of HIIT. The study population consisted of adults with obesity—defined by a Body Mass Index (BMI) ranging from 30 to 40 kg/m^2^—of both sexes, aged between 18 and 60 years, and who had not engaged in regular physical activity in the previous 6 months.

Only individuals who underwent clinical evaluation by the study's medical team and were deemed fit to participate in the exercise protocol were included. Participant recruitment took place between 25 October 2022, and 10 June 2024, and was conducted through the Community Extensive Sports Activities Program (PAEC) and the Nutrition Clinic database at Santo Amaro University (UNISA).

Exclusion criteria include individuals with diabetes using statins, HIV‐positive, Hepatitis B or C virus (HBV or HCV), and/or another systemic disease, undergoing treatment for obesity or bariatric surgery, participants in another regular exercise programme or severe motor disabilities, musculoskeletal injuries, severe respiratory diseases, heart disease, stroke or neurological disease or symptomatic cardiac malformations. And volunteers who attend less than 80% of the training days or who do not complete the training session on 3 consecutive days will be excluded from the study.

The sample size calculation was based on an effect size of 0.55, a significance level (α) of 5%, a confidence interval of 90% and a study power of 80%, reaching an n of 84 participants (G*Power, version 3.1.9.4). Group randomisation was performed through the Jerry Dallal online platform, available at: http://www.jerrydallal.com/random/randomise.htm.

### 2.2 Physical Exercise Protocol

The physical exercise programme was conducted over 8 weeks, with sessions held three times per week on a cycle ergometer (V3 Vertical Exercise Bike, Movement, Brazil). The protocol was designed to progressively increase both the duration and the number of high‐intensity intervals over time. Each session began and ended with a 5 min warm‐up, followed by a return to rest.

To allow for gradual adaptation, the training protocol varied weekly, following the protocol: Week 1: 4 bouts of 1‐min high‐intensity intervals (80%–100% of maximum heart rate [HRmax]), each followed by 3 min of active recovery (50%–70% HRmax). Week 2: 6 bouts of 1‐min high‐intensity intervals, each followed by 3 min of recovery. Week 3: 6 bouts of 1.5‐min high‐intensity intervals, each followed by 3 min of recovery. Weeks 4–8: 6 bouts of 2‐min high‐intensity intervals, each followed by 3 min of recovery.

Exercise intensity was continuously monitored using a Bluetooth‐enabled heart rate monitor (model H7, Polar, Finland), with data transmitted to the Polar Team application installed on a tablet or smartphone or recorded via a Polar watch. Target training zones were determined based on the estimated HRmax and verified using the Borg Rating of Perceived Exertion (RPE) scale to ensure that participants reached the prescribed intensity during each session.

### 2.3 Peripheral Mononuclear Blood Cells Sampling and Processing

Peripheral blood samples were collected in EDTA‐containing tubes to prevent coagulation. Approximately 15 mL of blood was obtained at baseline and again after 8 weeks of training. For participants in the trained group, postintervention blood collection was performed 24–48 h after the final HIIT session.

The samples were centrifuged at 800*g* for 10 min at room temperature to separate and remove excess plasma. The remaining content was diluted 1:1 with sterile phosphate‐buffered saline (PBS) and carefully layered onto Ficoll–Hypaque (Ficoll Paque Plus, GE Healthcare Bio‐Sciences AB, Uppsala, Sweden) in 15 mL Falcon tubes. Following centrifugation at 800*g* for 20 min at room temperature, the peripheral mononuclear blood cells (PBMCs) were collected, washed in PBS and centrifuged again under the same conditions.

After discarding the supernatant, 3 mL of ammonium chloride solution was added to the pellet for 2 min at room temperature to lyse residual red blood cells. The cells were then washed once more with sterile PBS and centrifuged (10 min at 800*g*, room temperature). Following removal of the supernatant, the pellet was resuspended in 1 mL of PBS, transferred to microtubes and subjected to a final centrifugation step. The resulting dry pellet was stored at −80°C until further analysis.

### 2.4 Determination of IL‐1β Concentration

Peripheral blood samples were collected under fasting conditions at two different time points: at baseline, before the intervention (T0), and after the 8‐week protocol (T1). For the trained group, samples were obtained 24–48 h after the last HIIT session. Blood was collected in appropriate tubes for serum separation, and after centrifugation at 2500 rpm for 10 min at 4°C, the serum aliquots were stored at −80°C for subsequent analysis of the inflammatory profile. The cytokine IL‐1β was quantified using a commercial ELISA kit (R&D Systems, USA), following the manufacturer's instructions. A *p* < 0.05 was considered statistically significant.

### 2.5 RNA Isolation and Reverse Transcription

To evaluate the transcription of the NLRP1 inflammasome and its associated components, RNA was extracted from PBMCs using the Trizol reagent, following the protocol described by Rangel et al. (2024) [19]. To eliminate potential genomic DNA contamination, the samples were treated with DNase using the Turbo DNA‐free kit (Thermo Fisher Scientific). RNA concentration and purity were assessed using a NanoDrop 2000 spectrophotometer (Applied Biosystems). Complementary DNA (cDNA) was synthesised via reverse transcription using the SuperScript II Reverse Transcriptase Kit (Thermo Fisher Scientific).

### 2.6 Gene Expression Analysis

As described by da Silva (2023) [20], each PCR reaction consisted of 10 μL of 2X TaqMan Gene Expression Master Mix (Thermo Fisher Scientific), DNase‐ and RNase‐free water, 1 μL of cDNA, and 1 μL of the corresponding 20X TaqMan Gene Expression Assay, totaling 20 μL per reaction. All reactions were run in duplicate and analysed using the StepOnePlus Real‐Time PCR System (Applied Biosystems). The primers used are detailed in Table 1. Gene expression levels were calculated using the 2^–ΔΔC*t*^ method, with GAPDH gene as the endogenous control (housekeeping gene) to normalise the data across samples. Results are reported as fold changes in gene expression relative to baseline.

**TABLE 1:** Genes and *primers* of the study.

Gene	*5′‐3′ Forward primer*	*5′‐3′ Reverse primer*
CARD18	CCAGAGCTTCTTCCAAGGGA	AAAGGAAGGGAAAGGGAGGG
CARD16	CCGAGCTTTGATTGACTCCG	GGCCCTAGGTGAACTTGAGT
IL‐1A	AGATGCCTGAGATACCCAAAACC	CCAAGCACACCCAGTAGTCT
AIM2	GCTGCACCAAAAGTCTCTCCTC	CTGCTTGCCTTCTTGGGTCTCA
MEFV	AGGAGCAGCGATCCTATGG	CAGCGCTTCAGTTTGTTTCA
NLRP1	CCACAACCCTCTGTCTACATTAC	GCCCCATCTAACCCATGCTTC
GAPDH	ACCCACTCCTCCACCTTTGAC	TGTTGCTGTAGCCAAATTCGTT

### 2.7 Gene Network Analysis

Differentially expressed genes (*p* < 0.05) were uploaded to the IntAct molecular interaction (MI) database [21] to identify potential gene interaction pathways. For quality control, only genes with direct interactions and associations were included in the analysis. Furthermore, interactions were filtered to include only those identified through methods such as ubiquitin reconstruction, antitag and anticoimmunoprecipitation (anti‐CoIP). Interactions with a MI confidence score greater than 0.35 were considered for inclusion.

### 2.8 Statistical Analysis

Descriptive measures and statistical tests were performed using SPSS software, version 23.0. The Shapiro–Wilk test was used to assess the normality of the variables, followed by the application of parametric or non‐parametric tests according to the type of variable. Comparisons between groups (Trained and Control) were made using the unpaired t‐test or Mann–Whitney test. The significance level was set at *p* < 0.05.

## 3 Results

**FIGURE 1:**
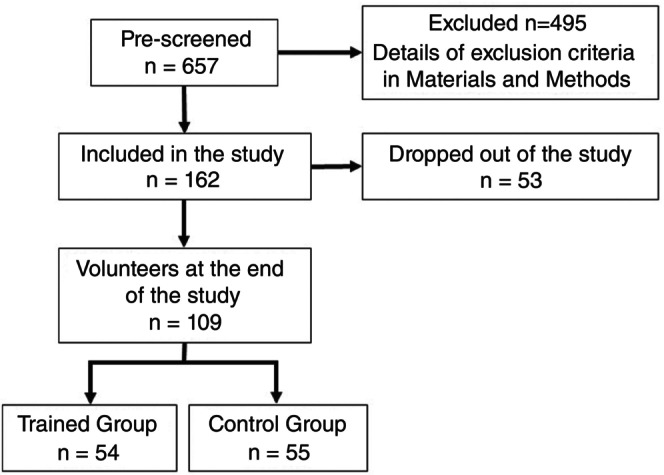
Flow chart illustrating the study design and main steps. Detailed flowchart of the study design, showing patient pre‐selection (*n* = 657), group allocation (Trained = 54; Control = 55), and two samples from each individual were collected throughout the study visits, T0 at beginning and T1 after 8 weeks.

Figure 1 shows the detailed flow chart of the study. Patients (*n* = 657) were preselected for clinical evaluation. Of these, 54 participants were included in the Trained group and 55 in the Control group. In the Control group, participants were instructed not to exercise for 8 weeks and samples were also taken on two visits.

Table 2 shows the general data of the participants. There were no statistically significant differences between the Control and Trained groups (baseline values) for any variable (all *p* > 0.05). The BMI values in the volunteers showed an average class I obesity (Trained group 34.14 kg/m^2^; Control group 34.34 kg/m^2^), which classifies an individual with an excessive amount of body fat for their height, which represents a health risk such as diabetes, hypertension and heart problems, as observed for both groups. Obesity is a condition of metabolic dysfunction and chronic inflammation, and this is reflected in changes in the expression of many genes in different tissues, especially those involved in the inflammatory process.

**TABLE 2:** General characteristics of the sample at the baseline visit.

	Trained group	Control group	*p*
*N*	54	55	
Female/male	46/8	44/11	0.48^a^
Age, years (mean ± SD)	45 (10)	44 (11)	0.81^b^
Weight, Kg (mean ± SD)	88.61 (15.43)	90.56 (13.29)	0.51^b^
Height, m (mean ± SD)	1.62 (0.09)	1.61 (0.07)	0.95^b^
BMI, Kg/m^2^ (mean ± SD)^$^	34.14 (3.58)	34.34 (3.85)	0.79^b^
Diabetes, *n* (%)	10 (19%)	11 (20%)	0.95^a^
Hypertension, *n* (%)	15 (28%)	11 (20%)	0.48^a^

Abbreviations: BMI, body mass index; SD, standard deviation.

^a^ Pearson Qui‐Square Test.

^b^ Unpaired T‐test.

### 3.1 Gene Expressions

The analyses considered the 2^−ΔΔC*t*^ method, which calculates the value of the fold change in gene transcription levels. A value of 1 corresponds to no change in gene expression, while values above 1 indicate an increase and below 1 a decrease in gene expression.

### 3.2 NLRP1, IL‐1A, AIM2, MEFV, CARD16 and CARD18 Expressions

**FIGURE 2:**
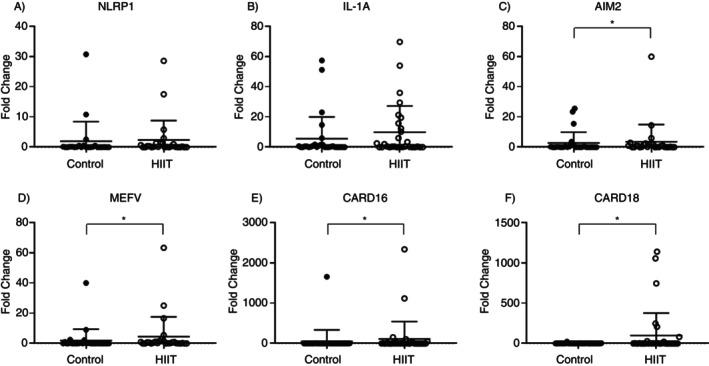
Expression of the genes (A) NLRP1 (NLR family pyrin domain containing 1), (B) IL‐1A (Interleukin‐1 alpha), (C) AIM2 (Absent In Melanoma 2), (D) MEFV (Mediterranean fever gene), (E) CARD16 (caspase recruitment domain family member 16) and (F) CARD18 (caspase recruitment domain family member 18). There was a significantly different increase (*p* < 0.05) in AIM2, MEFV, CARD16 and CARD18 in the Trained Group.

**FIGURE 3:**
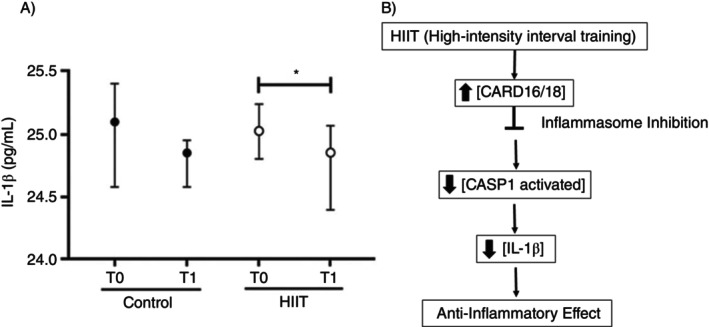
(A) Serum levels of IL‐1β in individuals subjected to high‐intensity interval training (trained group) compared with untrained individuals (control group). A significant reduction in IL‐1β was observed in the trained group (*p* < 0.05). (B) Schematic representation of the effects of high‐intensity interval training (HIIT) on inflammasome pathway modulation. HIIT induces an increase in CARD16/CARD18 expression, leading to the inhibition of inflammation through the reduction of active CASP1 and the consequent decrease in mature IL‐1β production, ultimately resulting in a systemic anti‐inflammatory effect.

After a comparison between the Trained and Control groups, there were no differences for NLRP1 and IL‐1A transcription levels, but an increase in AIM2, MEFV, CARD16 and CARD18 after 8 weeks of training (*p* < 0.05) (Figure 2). The Table S1 contain the comparisons between groups with their respective 95% confidence intervals (Table S1). Serum samples from patients undergoing HIIT were evaluated for circulating IL‐1β levels, and a significant decrease was observed in the trained group (*p* = 0.0129) (Figure 3A). While in Figure 3B, a representative scheme of the possible effects of HIIT on the modulation of the inflammasome pathway.

### 3.3 Genic Pathway Analysis

**FIGURE 4:**
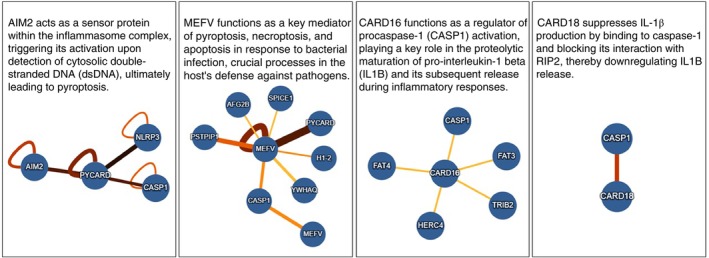
Genetic pathway and function of differentially expressed genes. Colour blue in the nodes represents the species *Homo sapiens* . Darker edges indicate a higher molecular interaction (MI) score, reflecting a greater probability that the interaction is accurate. Wider edges represent stronger experimental support for a given interaction.

Interaction analysis of differentially expressed genes (AIM2, MEFV, CARD16 and CARD18) revealed interactions with several genes, and some of these genes also interact with the NLRP1 inflammasome. The gene pathway of each differentially expressed gene and the function of each gene/protein (according to uniProt—https://www.uniprot.org/) are shown in Figure 4.

## 4 Discussion

Previous work by our group showed the impact of HIIT on the modulation of genes related to the NLRP3 inflammasome and its components, including inflammatory cytokines and chemokine receptors in monocytes, showing a significant reduction in the expression of CASP and IL‐6 genes, and an increase in the expression of the ASC gene in the Trained group (not published). Based on these findings, the current work evaluated the expression of genes related to the NLRP1, AIM2 and MEFV inflammasomes and genes downstream of the main pathways involved in inflammation, such as IL‐1A, CARD16 and CARD18. Genes directly related to ASC (PYCARD) were upregulated, such as AIM2 and MEFV. However, the trained group had a significant reduction in the expression of CASP, which is activated by the NLRP3 inflammasome, playing a central role in the maturation of the pro‐inflammatory cytokines IL‐1β and IL‐18. Therefore, its reduction may play an important role in reducing systemic inflammation [22, 23]. And despite the decrease in CASP expression and overexpression of ASC, there was no activation of the NLRP1 inflammasome with the activation of caspase‐1, which can be corroborated by the increased expression of the CARD16 gene, which according to our hypothesis supported by the transcription data, may be inhibiting caspase‐1 activation, preventing its dimerisation and reducing the maturation and release of IL‐1β [24]. In addition, the CARD18 gene also showed increased expression in the trained group compared to the untrained control group. Although the CARD18 gene interacts with the pro‐caspase‐1 CARD and auto aggregates, evidence regarding its function is still conflicting [25]. Evidence suggests that CARD18 may act in a negative feedback mechanism, reducing IL‐1β release by preventing RIP2 from binding to caspase‐1 [26]. In contrast, another study reported that CARD18, despite incorporating into caspase‐1, does not inhibit inflammasome activation [27]. Suggesting that this protein may have a double effect, being produced slowly so that the beneficial effects of inflammation can occur before the process is interrupted [13]. However, further studies in relevant cellular models are needed to determine the role of CARD18. Our results reinforce this perspective by showing that 8 weeks of HIIT modulate key components of the inflammasome, suggesting that physical exercise may contribute to regulating gene transcription and thus CARD18 activity.

Of the genes that were not modulated, IL‐1A is known to be constitutively present as a precursor in all healthy tissues with barrier function, and IL‐1A precursor production can be induced in myeloid cells during inflammation, but generally requires intense stimuli from tissue injury or infection to be elevated detectably [28]. The type of response triggered by HIIT was likely insufficient to alter IL‐1A gene expression. Therefore, it is plausible that HIIT, despite causing metabolic adaptations and modulation of other inflammatory components, did not generate the degree of cellular damage or danger signalling necessary to significantly alter IL‐1A gene expression.

The pathogenesis of disorders resulting from constitutive activation of inflammasomes leads to increased caspase‐1 activity, IL‐1β and IL‐18 release, and pyroptosis [29]. Currently in clinical practice, there are three IL‐1 inhibitors used to treat inflammasome‐related disorders: anakinra (Kineret), a recombinant version of the human IL‐1R antagonist protein (IL‐1Ra), canakinumab (Ilaris), a human monoclonal antibody that targets IL‐1β and rilonacept (Arcalyst), a fusion of the human IL‐1R component (IL‐1R1) and the IL‐1R accessory protein (IL‐1RAcP) that potently binds to IL‐1β and IL‐1α [28]. Aiming to promote well‐being and potentially reduce the patient's pharmacological intake, HIIT, like practice physical exercises, has been shown to be effective in reducing inflammatory markers in some studies. However, intensity and duration may be crucial factors in achieving more robust effects, which need to be adjusted. Thus, HIIT has been shown to be a promising intervention in modulating inflammatory processes, especially those associated with obesity [30]. Previous studies have shown that a 6‐month aerobic exercise intervention neutralised inflammasome activation, significantly improving insulin sensitivity and several metabolic markers in obese individuals [30]. This leads to beneficial effects on the body, thereby inhibiting or delaying inflammasome activation.

Butts et al. (2018) [31] evaluated physical exercise against the increase in the mean percentage of ASC methylation and the decrease of IL‐1β and ASC gene expression in heart failure, linking the epigenetic regulation of ASC as a biological mechanism by which physical exercise can promote better outcomes in heart failure. In another study, Barrón‐Cabrera et al. (2020) [32] investigated the effects of moderate‐intensity physical exercise for 4 months combined with diet in obese individuals and found a reduction in ASC expression in the group that received the diet plus exercise combination compared to the group that received diet alone. This fact corroborates the importance of physical exercise, whether combined or not with diet, to ensure an improvement in systemic inflammation.

Inflammasome activation is a key function mediated by the innate immune system, and recent advances have greatly increased our understanding of macromolecular inflammasome activation. Analysis of gene expression pathways revealed several genes that may be regulated by the NLRP1, AIM2 and MEFV (pyrin) inflammasomes. These genes are associated with immune response, inflammation, apoptosis, activation, and structural proteins (cytoskeleton, membrane and endosome formation). Although the NLRP1 inflammasome can be found in most cell types and tissues, it appears to play a vital role in epithelial barriers, particularly in the integumentary and respiratory systems [33], which can be a challenge for future studies. The NLRP1 inflammasome also interacts with PYCARD‐1 (ASC), BCL2 and BCLL1, among other genes (Figure 3B). Upon sensing certain stimuli, AIM2 can oligomerise to become a caspase‐1 activating scaffold. Active caspase‐1 subsequently functions to cleave the IL‐1 family of proinflammatory cytokines into their bioactive forms, IL‐1β and IL‐18, and cause pyroptosis, a type of inflammatory cell death [34]. The AIM2 inflammasome also interacts with caspase‐1 via PYCARD (ASC) and, in the context studied, is upregulated. When activated, this inflammasome contributes to the immune response against bacterial and viral infections by binding directly to dsDNA [35].

The MEFV gene encodes the pyrin only protein, found primarily in immune cells such as neutrophils, monocytes and dendritic cells. Pyrin is a unique immune sensor because it detects bacterial virulence through cytoskeletal remodelling rather than microbial compounds [36]. The pyrin inflammasome (MEFV) was upregulated, and like the other two, it is also capable of activating caspase‐1 via ASC, triggering its inflammatory effects [37]. Regarding the genes involved in inflammasome signalling, we evaluated the transcription of the genes CARD16 and CARD18, both of which were upregulated. Our hypothesis, even based on the increase in the transcript of CARD16, is that in its native protein form, competitively binds to caspase‐1, preventing its dimerisation and reducing the maturation and release of IL‐1β [38]. And similarly, CARD18 could also be interacting with caspase‐1, although evidence about its function is still conflicting and ambiguous [26, 39].

Humke et al. showed that CARD18 can be induced by pro‐inflammatory stimuli and binds directly to pro‐caspase‐1, preventing its association with the adaptor RIP2. This interaction would block caspase‐1 activation and, consequently, reduce the cleavage and release of IL‐1β, the main cytokine of the inflammasome. Gain‐of‐function experiments showed that CARD18 overexpression robustly suppresses IL‐1β production, confirming its inhibitory action. These findings marked the identification of a negative feedback mechanism that limits excessive inflammatory responses, significantly expanding our understanding of the molecular control of inflammasome activation and innate immunity [26].

The results of this study suggest that HIIT may induce an acute and transient inflammatory response, characterised by the initial activation of genes related to inflammasomes, such as AIM2 and MEFV. However, this activation is accompanied by increased expression of negative regulatory genes, such as CARD16 and CARD18, which appear to act as feedback mechanisms preventing the exacerbation of the inflammatory process. Thus, HIIT does not promote chronic inflammation but rather a temporary inflammatory stimulus that triggers beneficial metabolic and immunological adaptations, contributing to the resolution of inflammation and the improvement of the low‐grade inflammatory state associated with obesity.

Aberrant inflammasome signalling has been implicated in the development of cardiovascular and metabolic diseases, cancer and neurodegenerative disorders. Obesity, a chronic disease, is associated with a systemic inflammatory state, which, along with elevated cytokine production, mediates the activation of the intracellular protein complex called inflammasomes. Therefore, understanding how the inflammasome complex matures and its subsequent signalling is crucial for developing appropriate therapies and interventions for each pathological disorder.

This study advances previous work by more comprehensively exploring the link between physical exercise and inflammasome modulation. Until now, most research has focused on NLRP3, often associated with exacerbated inflammatory responses and metabolic diseases. By including NLRP1, AIM2 and MEFV, this work broadens the scope of investigation, bringing new insights into the possible inhibitory role of CARD16 and CARD18 in the context of physical activity.

Finally, it is important to emphasise that, although this study presents relevant strengths, such as the rigorous recruitment and selection process of volunteers, the controlled execution of the HIIT protocol, and the analysis of the expression of genes related to inflammasomes (NLRP1, AIM2 and MEFV), some limitations should be considered. Among these, the number of participants in each group stands out, which, while sufficient to meet the proposed objectives, may restrict the extrapolation of the results to the general obese population. Furthermore, external factors, such as dietary habits, use of supplements (vitamins and antioxidants), sleep patterns, stress and physical activity levels outside the protocol, were not strictly controlled. Another aspect to consider is the applicability of the findings, which may be limited in different populations, such as the elderly, individuals with severe obesity or those with specific comorbidities. These limitations, however, open perspectives for future studies that contemplate greater control of external variables and incorporate complementary functional and clinical analyses, expanding the understanding of the effects of HIIT on modulating inflammation.

## 5 Conclusions

Inflammation is an essential and beneficial physiological process, fundamental for the elimination of pathogens and the promotion of tissue repair after injury. However, when it becomes chronic or dysregulated, inflammation exerts harmful effects, contributing to the onset and progression of various systemic inflammatory diseases. Therefore, there is growing interest in understanding the molecular mechanisms that regulate the inflammatory response, aiming to enable the development of new therapeutic approaches for pharmacological inflammation control.

Based on the findings of this study, it can be concluded that HIIT plays a relevant role in modulating the gene expression of inflammasome‐related components, especially AIM2 and MEFV, as well as downstream genes involved in the regulation of inflammation, such as CARD16 and CARD18. The upregulation of genes such as CARD16 and CARD18 points to possible inhibitory feedback mechanisms on caspase‐1 activation, although the function of CARD18 remains controversial and requires further study. The reduction in CASP expression, combined with the increase in ASC expression, suggests a protective effect of physical exercise against the exacerbated activation of caspase‐1 and, consequently, on the maturation and release of pro‐inflammatory cytokines such as IL‐1β, a reduction also detected in the serum of trained volunteers. Overall, the data reinforce the importance of physical exercise as a complementary strategy in managing inflammation in obese individuals, opening perspectives for future investigations.

## Author Contributions

A.L.P.A.S., D.A.A., A.L.S.M. and M.T.S. have made substantial contributions to the conception, design, acquisition and analysis of the data. L.H.S.N., J.B.A., A.L.L.B., P.R‐.T. and C.N.F. have made substantial contributions to the conception, design, draft of the work and revision of the work. All authors read and approved the final manuscript.

## Funding

This work was supported by the Fundação de Amparo à Pesquisa do Estado de São Paulo (2021/12190‐5, 2023/18019‐1, 2022/13905‐0) and the Conselho Nacional de Desenvolvimento Científico e Tecnológico (302816/2021‐6).

## Ethics Statement

Participants read and signed the free and informed written consent form (TCLE). The study was approved by the Research Ethics Committee of the University of Santo Amaro (number 5.995.573), registered as a clinical trial before its start (Brazilian Registry of Clinical Trials—ReBEC number RBR‐8vfxfqd) and followed the principles described in the Declaration of Helsinki.

## Consent

Participants read and signed the free and informed written consent form (TCLE).

## Conflicts of Interest

The authors declare no conflicts of interest.

## Supporting information


**Table S1:** Comparison between control and trained groups after 8 weeks of HIIT.

## Data Availability

The data that support the findings of this study are available on request from the corresponding author. The data are not publicly available due to privacy or ethical restrictions.
