# Supplementary material for: The Role of NLRP1, AIM2 and MEFV Inflammasomes in the High‐Intensity Interval Training of Individuals With Obesity
Source: Immunology. 2025 Dec 21;178(1):109–17. doi: 10.1111/imm.70090 (PMC13079245; doi:10.1111/imm.70090)
Supplement: Supplementary file 1 — Table S1: Comparison between control and trained groups after 8 weeks of HIIT. [file IMM-178-109-s001.docx]

## **Supplementary Table 1.** Comparison between Control and Trained groups after 8 weeks of HIIT

|  | **Control Group**  **(mean)** | **Trained Group**  **(mean)** | **Difference**  **(95% CI)** | **p-value^#^** |
| --- | --- | --- | --- | --- |
| CARD18 | 1.33 | 98.60 | 97.26 (0,3364 to 194,2) | 0.0005 |
| CARD16 | 49.43 | 111.5 | 62.07 (-113,6 to237,8) | 0.005 |
| MEFV | 1.94 | 4.57 | 2.63 (-3,004 to 8,268) | 0.02 |
| AIM2 | 2.80 | 3.58 | 0.78 (-4,442 to 6,016) | 0.03 |
| NLRP1 | 1.93 | 2.39 | 0.46 (-3,20 to 4,13) | ns |
| IL-1A | 5.59 | 9.81 | 4.21 (-4,19 to 12,62) | ns |

^#^ Mann-Whitney test. ns: not significant.
